# A brief discussion on the role of calcitonin gene-related peptide in the efficacy of rehabilitation medicine

**DOI:** 10.3389/fresc.2025.1593487

**Published:** 2025-06-09

**Authors:** Guan Zhencheng, Xue Aiguo

**Affiliations:** ^1^ Department of Acupuncture, Dongguan Hospital of Traditional Chinese Medicine, Dongguan, Guangdong, China

**Keywords:** calcitonin gene-related peptide (CGRP), physical and rehabilitation medicine, pain management, neuroprotection, vasodilation

## Abstract

Calcitonin gene-related peptide (CGRP) is an active peptide composed of 37 amino acids that functions through specific receptors. It is widely distributed in small-diameter dorsal root ganglion neurons, trigeminal ganglion neurons, and nerve fibers innervating the spinal cord and brainstem dorsal horn. CGRP regulates various physiological functions, including vasodilation, inflammation modulation, and cardiac protection, and plays a key role in pain transmission. Pain is a global health challenge closely associated with the activity of neuropeptides such as CGRP. Although progress has been made in the application of CGRP in treating various diseases, research in the field of rehabilitation remains in its early stages. This article summarizes the roles of CGRP in peripheral nerve injury, central injury, cardiovascular rehabilitation, and pain rehabilitation. In terms of treatment, common physical therapies such as laser therapy and shock wave therapy have been shown to influence CGRP expression levels. However, the specific effects of these physical interventions on CGRP require more systematic future research and analysis to achieve more efficient and personalized rehabilitation strategies.

## 1 Introduction

Pain, a global health burden and rehabilitation challenge, disrupts biopsychosocial functioning through multifactorial pathophysiology involving cellular signaling networks. Neuropeptides critically engage receptors and inflammatory mediators in nociceptive processing, driving persistent pain states.

Current research advancements in neurogenic inflammation and pain-related disorders, including migraine and arthritic conditions, have established calcitonin gene-related peptide (CGRP) as a crucial neuromodulator in pain pathophysiology. Extensive scientific evidence demonstrates that CGRP functions as both a key mediator and amplifier in nociceptive transmission pathways, with its expression levels directly correlating with pain intensity and duration across various clinical manifestations (1). This review examines CGRP's dual roles in pain pathophysiology and its translational potential as a therapeutic target in precision rehabilitation medicine.

Calcitonin gene-related peptide (CGRP) is an active polypeptide composed of 37 amino acids, formed through specific splicing of the calcitonin gene (CALCA). In humans, CGRP exists in α and β forms, which share 94% structural similarity. CGRP binds to specific receptors and is widely distributed in the body, particularly in small-diameter dorsal root ganglion neurons, trigeminal ganglion neurons, and nerve fibers controlling the spinal cord and brainstem dorsal horn. α-CGRP and β-CGRP exhibit similar biological activities, with α-CGRP being the predominant form in the central and peripheral nervous systems, while β-CGRP plays a more significant role in intestinal function. The expression of calcitonin and CGRP mRNA is tissue-specific. Calcitonin (CT), a well-known peptide hormone isolated from the parathyroid gland, is involved in calcium ion homeostasis. Early studies on CT gene cloning revealed alternative RNA processing in neuronal tissues, leading to the synthesis of mRNA encoding CGRP (2).CGRP mRNA is synthesized by splicing the first three exons of CALCA to the fifth and sixth exons, followed by post-translational modifications and proteolytic cleavage to form the mature peptide (3–5). CGRP's pleiotropic activity encompasses vasodilation, immunomodulation, cardioprotection, and nociceptive signaling.

## 2 Functions and mechanisms of CGRP

### 2.1 Advances in basic medical research on CGRP

CGRP critically mediates nociceptive signaling, facilitating pain transmission from peripheral nociceptors to the amygdala via spinal dorsal horn release during inflammatory and noxious stimuli. In chronic pain states, CGRP upregulation in sensory neurons—particularly C fibers (mediating chronic dull pain) and Aδ fibers (processing acute sharp pain)—drives peripheral and central sensitization mechanisms (6–8). CGRP potentiates central sensory neuron excitability, augmenting spinal nociceptive transmission and driving central sensitization in chronic pain. Arthritis models demonstrate amplified synaptic facilitation by CGRP, contrasting its dual modulation (excitatory/inhibitory) in physiological states (6). CGRP signaling critically mediates visceral pain, notably bladder-colonic cross-sensitization in pelvic pain pathways, as evidenced by preclinical models (7, 9). Emerging evidence links spleen deficiency-associated functional dyspepsia to dysregulated motility-modulating brain-gut peptides, particularly CGRP (10). In murine TMD models with joint inflammation and masticatory muscle injury, TRPV4 activation in trigeminal ganglion neurons drives CGRP release, suggesting therapeutic potential of TRPV4/CGRP blockade for TMD pain (11).

In rat OA pain models, CGRP antibody blockade alleviated pain with sustained (>60 days) analgesia in NSAID-refractory cases, demonstrating therapeutic potential across OA pain subtypes (12).Pota et al. found that short-term exposure to 17β-estradiol increased CGRP release in F11 cell lines at 200 mg/kg, highlighting the role of sex hormones in pain transmission and potential explanations for sex differences in pain sensitivity (13).

Additionally, the dual role of CGRP in inflammation modulation is increasingly recognized. In a rat asthma model, CGRP released by pulmonary neuroendocrine cells (PNECs) enhances ILC2 activity, leading to airway smooth muscle contraction and mucus cell hyperplasia, exacerbating allergic responses (14, 15).

CGRP, the potent vasodilator central to migraine pathophysiology, induces migraine-like attacks when administered exogenously. Anti-CGRP therapies demonstrate dual efficacy in acute and prophylactic migraine management without triptan-like vasoconstriction, supported by consistently positive clinical trial outcomes and favorable safety profiles (2, 14, 16–19). While propelling anti-CGRP drug development, a 1994 study paradoxically found spinal CGRP administration failed to alter nociceptive responses, highlighting methodological discrepancies (model systems, dosing, nociceptive assays) underlying outcome variability (16).

CGRP exerts therapeutic potential in cardiovascular disease through its potent vasodilatory effects, enhancing regional perfusion to confer adjuvant benefits in hypertensive and coronary conditions. Crucially, CGRP antagonizes multiple vasoconstrictors (serotonin, endothelin, neuropeptide Y) while promoting hemodynamic optimization and cardioprotection in cardiovascular pathologies (20, 21).

Beyond neurodegeneration, CGRP exhibits neuroprotective potential via PKA-mediated hippocampal metabolic regulation and anti-inflammatory mechanisms, positioning it as an emerging therapeutic target in Alzheimer's disease research (22).

Denervation can lead to bone density changes, occasionally progressing to neurogenic osteoporosis. Studies confirm that CGRP stimulates bone formation. Bone density maintenance relies on intact innervation, and CGRP directly or indirectly modulates osteoporotic processes (23, 24). Both osteoblasts and osteoclasts express CGRP receptors, enabling CGRP to promote bone formation and inhibit resorption. In mice with CGRP gene overexpression, trabecular bone density increased, alleviating osteoporosis (25, 26).

Over the past decades, CGRP's roles in digestive, nervous, cardiovascular, skeletal, and respiratory systems have been extensively studied, with clinical advances in CGRP antagonists. However, the biological functions and therapeutic potential of CGRP in rehabilitation medicine remain underexplored.

### 2.2 Potential roles of CGRP in rehabilitation medicine

CGRP's applications in rehabilitation are not yet mainstream due to limited research on its direct links to rehabilitation-related dysfunctions. Nevertheless, understanding CGRP's distribution and mechanisms can inform future rehabilitation strategies. Further research may enhance pain biology understanding and offer new approaches for chronic pain management, improving patients’ quality of life and rehabilitation outcomes.

#### 2.2.1 Role of CGRP in peripheral nerve injury rehabilitation

CGRP is highly enriched in spinal dorsal horn and primary afferent fibers, serving as a neuroregeneration biomarker. Following nerve injury, its upregulated expression triggers p-CREB/c-fos signaling cascades that enhance neuroplasticity, as evidenced by massage-induced CGRP modulation in rat sciatic injury models where dorsal root ganglia alterations correlate with spinal cord repair (27).

Post-injury upregulation of CGRP in DRG neurons exhibits pain-associated neuroinflammatory effects. Neural mobilization therapy in rabbit sciatic injury models demonstrated DRG CGRP downregulation, attenuating neuroinflammation, suppressing central sensitization, and enhancing functional recovery (28). Thus, CGRP has dual roles in peripheral nerve injury: promoting nerve survival and regeneration while potentially exacerbating acute inflammation.

#### 2.2.2 Role of CGRP in central injury rehabilitation

Emerging evidence implicates CGRP in acupuncture and neurorehabilitation mechanisms for CNS injuries. In rat ischemic stroke models, CGRP/NGF co-administration suppressed pro-apoptotic mediators (ICAM-1/Fas), exerting neuroprotection via dual anti-inflammatory and anti-apoptotic pathways (29). GV26 electroacupuncture modulated neurovascular homeostasis in cerebral ischemia by elevating CGRP and downregulating AVP/Ang-II (21). Multimodal early rehabilitation (sensorimotor stimulation, environmental enrichment) in neonatal HIBI models upregulated pan-tissue CGRP expression, correlating with enhanced neuromotor recovery and cognitive improvement (30). In rodent TBI models, CGRP attenuated edema, regulated autophagic-apoptotic crosstalk through Akt/mTOR axis modulation, and mitigated neuroinflammatory responses (31). CGRP upregulation in cerebral ischemia predicts survival, implicating preconditioning-mediated ischemia tolerance (32).

Experimental investigations in spinal cord injury (SCI) models have revealed dense CGRP-positive fiber proliferation within the deeper laminae of the dorsal horn post-injury. This pathological sprouting potentiates maladaptive sensory afferent recruitment in segmental spinal reflex circuits, thereby exacerbating autonomic dysreflexia (AD)—a life-threatening autonomic dysfunction. Given the conserved neuroanatomical reorganization patterns between animal models and human SCI pathophysiology, these findings underscore a paradoxical risk: therapeutic strategies aimed at neurorestoration may inadvertently promote AD pathogenesis through aberrant circuit reinnervation (33).

#### 2.2.3 Role of CGRP in cardiovascular rehabilitation

CGRP-positive fibers innervate atrial/ventricular myocardium, coronary vasculature, and cardiac conduction systems. Rodent studies reveal endurance training upregulates plasma/cardiac CGRP, contributing to physiological cardiac remodeling. In contrast, exhaustive exercise suppressed DRG CGRP synthesis, while exercise preconditioning preserved CGRP levels to promote cardioprotective adaptation (20). CGRP exerts cerebroprotective effects against diabetic cerebrovascular remodeling via triple mechanisms: antagonizing AngII-induced hypertension, suppressing apoptosis, and mitigating oxidative stress (32).

As previously discussed, CGRP exerts potent vasodilatory effects and can reduce blood pressure by modulating peripheral resistance. However, in a phenol-induced hypertensive animal model, while administration of an α2 receptor antagonist restored the reduced CGRP levels, it failed to lower blood pressure—indicating a loss of CGRP's blood pressure regulatory capacity. The authors propose that α2 receptor-mediated enhancement of sympathetic tone amplifies pressor effects, thereby counterbalancing CGRP's blood pressure-lowering activity (1).

#### 2.2.4 Role of CGRP in pain rehabilitation

Myofascial pain is commonly associated with trigger points and elevated substance P, CGRP, and bradykinin (34). Manual therapy in rodent myofascial pain models attenuated nociceptive neuropeptides (CGRP/SP), alleviating neuromuscular hyperactivity and tissue rigidity (35). Degenerated IVDs in chronic LBP drive pathological crosstalk by releasing inflammatory mediators (TNF-α, IL-1β) and neurotrophins (NGF/BDNF) that upregulate DRG neuronal CGRP (36). Meta-analyses implicate CGRP elevation across multimodal pain states (somatic/visceral/neuropathic). Clinical evidence shows marked CGRP increases in OA synovial fluid, whiplash syndrome, and post-disc herniation cases, with elevated serum/synovial levels correlating with pain severity (37–39). CGRP antagonists inhibit capsaicin-induced dermal blood flow increases, supporting their role in pain modulation (40). Mechanical stress upregulates CGRP in degenerative IVDs, potentially contributing to chronic discogenic pain (41).

#### 2.2.5 Effects of physical therapies on CGRP Expression

Photobiomodulation therapies (LLLT/LED) differentially modulate neuropeptide release; 808-nm LLLT selectively upregulated SP without affecting CGRP in rodent dermal models (42). Conversely, LLLT elevated CGRP levels in gingival crevicular fluid (43). In a rat sciatic nerve injury model, low-power laser initially increased CGRP expression, which later declined as physical interventions dominated (44).

In rodent models, Extracorporeal Shock Wave Therapy dose-dependently attenuated epidermal CGRP-positive fibers, with parallel reductions in DRG and spinal cord CGRP levels, implicating peripheral-central nociceptive modulation (45).

In fructose-induced metabolic syndrome models, chronic fructose exposure suppressed CGRP levels, while 2-week TENS intervention elevated CGRP by 14.5% to normal levels alongside metabolic improvements, indicating CGRP's critical role in TENS-mediated sympathetic modulation (46).

Exercise therapy studies reveal paradoxical effects: treadmill-exercised rats showed amplified migraine susceptibility via CGRP upregulation, suggesting exercise-induced CGRP elevation may increase pain sensitivity. This necessitates cautious exercise prescription for migraine-prone patients (47).

### 3.1 Research prospects of CGRP in rehabilitation medicine

While mechanistic insights advance, CGRP research remains preclinical (rodent-focused) with human translational gaps persisting in functional rehabilitation. Combination therapies require rigorous validation for sustained efficacy.

For example, I would like to raise a doubt here. TENS, as a recognized physiotherapy for pain relief, should be more inclined to reduce the level of CGRP to reduce the sensitization of pain pathways, but for some studies, its level increases (46, 48). Whether there are other influencing mechanisms needs more verification. And there is another research of exercise therapy suggest the opposite than the other (e.g., low-intensity muscle contraction reduces CGRP in a arthritis research (49), while high-intensity running increases it (47), perhaps the effects of different forms of exercise on CGRP (especially in humans rather than rats) need to be explored.

In chronic pain management, physical modalities (thermal/mechanical) may exert CGRP-mediated analgesia, though systematic clinical substantiation remains imperative. Notably, psychoneuroimmunological interactions may potentiate CGRP modulation, optimizing rehabilitation paradigms (50).

Current rehab modalities (electrotherapy/ultrasound) lack CGRP mechanistic insights. Elucidating CGRP-pathway modulation could enable precision rehab protocols through biomarker-guided intervention strategies.

Priority research axes should dissect CGRP's disease-specific mechanisms and quality-of-life impacts to develop combined physio-pharmacological strategies for personalized therapeutic optimization.

### 3.2 Conclusion

Calcitonin gene-related peptide (CGRP), a pivotal neuropeptide in humans and mammals, plays a central regulatory role in pain processing, neurological functions, and cardiovascular homeostasis. These physiological processes hold direct clinical relevance to rehabilitation medicine, particularly in conditions requiring clinical interventions such as musculoskeletal pain syndromes, post-spinal cord injury rehabilitation (including motor function recovery and chronic pain management), and cardiovascular rehabilitation. Emerging evidence suggests that the pathophysiological mechanisms underlying these disorders are intricately linked to sensory neural pathways, thereby implicating CGRP signaling in their pathogenesis and therapeutic management.

This brief discussion talks about CGRP-mediated mechanisms in disease etiology and treatment response. Significantly, it proposes a novel conceptual framework for understanding how physiotherapeutic approaches might exert their therapeutic effects through modulation of CGRP expression—a hypothesis that warrants empirical validation through targeted mechanistic studies. Such investigation could potentially reveal new dimensions in optimizing rehabilitation protocols through neuropeptide pathway regulation.
